# The Cyclopœdia of Practical Medicine

**Published:** 1844-06

**Authors:** 


ART. V.—*The Cyclopædia of Practical Medicine.* Edited by JOHN FORBES, M.D., F.R.S., ALEX. TWEEDIE, M.D., F.R.S., and JOHN CONOLLY, M.D. *Revised, with additions*, by ROBLEY DUNGLISON, M.D. Philad: Lea & Blanchard. 1844. Part I. To be completed in 24 parts; a part to be issued every 2 weeks. Price 50 cents a number.

This Cyclopædia is pronounced, on all hands, to be one of the most valuable of the medical publications of the day. It is meant to be a library of practical medicine. As a work of reference, it will be invaluable. Among the contributors to its pages it includes many of the most experienced and learned physicians of the age, and although it must happen in such a work that some topics will not be treated with sufficient fullness, while others may occupy undue space, as a whole it forms a compendium of medical science and practice from which practitioners and students may draw the richest instruction. Y.

